# Incidental Extracardiac Findings of Technetium-99m Pyrophosphate Scintigraphy: A Pictorial Review

**DOI:** 10.7759/cureus.62316

**Published:** 2024-06-13

**Authors:** Ahmed L Fathala

**Affiliations:** 1 Radiology, King Faisal Specialist Hospital and Research Center, Riyadh, SAU

**Keywords:** spect, planar imaging, tc-99m pyp imaging, incidental noncardiac findings, cardiac amyloidosis, nuclear cardiology

## Abstract

Technetium-99m pyrophosphate (Tc-99m PYP) cardiac imaging is a simple, widely available, noninvasive method to identify patients with transthyretin-type cardiac amyloidosis (ATTR), and it has remarkably high diagnostic accuracy with very high sensitivity and specificity. Visual scores of 0, 1, 2, and 3 indicate non-myocardial uptake, uptake less than rib, equal to rib, and greater than rib uptake, respectively. Semiquantitative assessment using the heart-to-contralateral lung ratio of more than 1.5 at 1 hour accurately distinguishes ATTR from the cardiac amyloid light chain subtype. However, there are several incidental non-cardiac findings that can be seen in planar images, rotating single-photon emission computed tomography (SPECT) images, maximum intensity projection images, or computed tomography images acquired for attenuation correction. These findings may lead to the early detection of a noncardiac condition that may require additional treatment. The intent of this review is to demonstrate several incidental noncardiac abnormalities that have an impact on patient management and follow-up.

## Introduction and background

Cardiac amyloidosis is a disease characterized by extracellular deposition of fibrillary protein, and it is an underdiagnosed cause of heart failure and restrictive cardiomyopathy [1]. The two types of cardiac amyloidosis are transthyretin-related amyloidosis (ATTR) and cardiac amyloid light chain (AL) amyloidosis. The AL subtype is a clonal plasma cell disorder that eventually leads to the deposition of immunoglobulin light chains in the tissue causing organ dysfunction. ATTR involves misfolded monomers or dimers of normally tetrameric transthyretin protein (TTR) from either mutant TTR (also known as ATTRm, causing familial amyloid cardiomyopathy) or wild-type TTR (causing senile systemic amyloidosis (SSA)) deposited in the myocardium [2]. Detecting cardiac amyloidosis is difficult because patients present with nonspecific symptoms, nonspecific electrocardiography, and echocardiography findings. The clinical presentation of amyloid infiltration of the cardiovascular system is wide, ranging from asymptomatic atrioventricular and bundle-branch block to severe rapidly progressive heart failure owing to restrictive cardiomyopathy. It is important to identify patients with cardiac amyloidosis at an early stage to initiate appropriate therapy, and it is crucial to differentiate between AL and ATTR subtypes due to significantly different outcomes and management. Cardiac AL amyloids are typically amenable to chemotherapy but cardiac ATTR amyloidosis may benefit from novel TTR-specific treatment [3,4]. Previously, it was thought that no effective therapy existed for cardiac amyloidosis; however, this is no longer the case. Patients with the AL type of cardiac amyloidosis may have improved survival for up to 12 years with appropriate chemotherapy [5]. Furthermore, novel pharmacological agents are available and under development for the ATTR subtype [6]. In recent years, radionuclide scintigraphy with bone-seeking tracers, such as technetium-99m (Tc-99m)-diphosphono-1,2-propanodicarboxylic acid, Tc-99m-pyrophosphate (Tc-99m PYP), and Tc-99m-hydroxymethylene diphosphonate, has emerged as a valuable tool in the diagnosis of cardiac amyloidosis subtypes. There are two methods to report a TC-99m PYP scan: semiquantitative visual scoring and quantitative analysis. Semiquantitative visual scoring is as follows: 0 = indicated absent myocardial uptake, 1 = mild myocardial uptake less than bone, 2= moderate myocardial uptake equal to bone, and 3 = intense myocardial uptake greater than bone with mild or absent bone uptake. Recent multicenter studies have demonstrated greater than 90% sensitivity and specificity of bone scintigraphy in distinguishing between ATTR and AL subtypes [7,8]. The actual mechanism by which bone-seeking tracers visualize cardiac amyloidosis remains unclear and debatable [9].

Most experience in imaging protocols for Tc-99 m PYP is with combined planar and single-photon emission computed tomography (SPECT) imaging of the chest or planar imaging followed by SPECT if planar imaging is positive [10]. Whole-body TC-99m PYP did not provide additional diagnostic information to confirm cardiac amyloidosis. In addition to the evaluation of cardiac uptake of PYP to diagnose cardiac amyloidosis, any unexpected extracardiac (e.g., skeletal and soft tissue) radiotracer uptake must be reported as well by the interpreting physician. Planar imaging of extracardiac radiotracer uptake is limited by a lack of anatomic correlation and localization. SPECT imaging provides three-dimensional information about the depth and spatial localization of activity. Hybrid SPECT/computed tomography (CT) allows precise localization of the abnormal uptake and can improve specificity by distinguishing benign from malignant and nonosseous lesions [11]. However, SPECT/CT requires additional imaging time and delivers radiation to the patient, so it should only be used when necessary to aid image interpretation [12]. The true incidence and clinical significance of extracardiac TC-99m PYP uptake are unknown, and scant data are found in the literature. Extracardiac findings have been reported in breast tissue, bones and joints, and the lung [13]. Detection of extracardiac findings, such as cancer (e.g., lung cancer), in patients with cardiac amyloidosis could affect patient care drastically [14]. Therefore, the main objectives of this pictorial assay are to review the findings about extracardiac uptake of PYP in both planar and SPECT images and to review the use of CT attenuation correction (CTAC) scans from SPECT/CT in patients referred for TC-99m PYP imaging for suspicion of cardiac amyloidosis.

## Review

Findings on planar and SPECT images

Musculoskeletal Uptake

**Figure 1:**
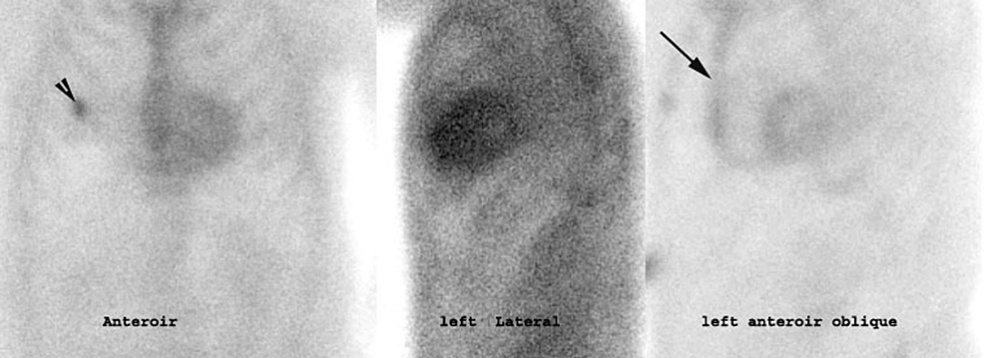
Anterior, left lateral, and left lateral oblique planar images of technetium-99m pyrophosphate (Tc-99m PYP) scintigraphy Anterior, left lateral, and left lateral oblique planar images of technetium-99m pyrophosphate (Tc-99m PYP) scintigraphy showing diffuse sternal uptake (arrow) and focal uptake in the right rib cage consistent with previous right rib fracture (arrowhead); in the left anterior oblique view, the appearance of the sternal uptake mimics uptake in the right ventricle

The thoracic region includes the sternum, clavicle, scapula, and ribs. It is important to recognize normal variants in the thoracic spine, as they can mimic pathology. Symmetrical uptake in the sternoclavicular joints is common in patients with degenerative joint disease [15]. Tracer uptake at the manubriosternal junction is a common, nonspecific finding commonly considered a normal variant. An eccentric, irregular, asymmetrical focal uptake situated distant from the manubrium is suspicious for a metastatic lesion [16]. Symmetrical tracer uptake in bilateral acromioclavicular and sternoclavicular joints is typically related to age-realized degenerative changes; occasionally, the tip of the scapula overlying a rib may mimic a focal abnormality. Therefore, it is useful to take an additional view with the arm raised to move the scapula outside the line of the ribs. A vertical, linear increase in uptake in the sternum is seen in patients who underwent sternotomy (e.g., for coronary artery bypass grafting or valve replacement surgery); on a lateral view of the chest, the linear activity in the sternum may mimic a dilated right ventricle (Figure 1).

**Figure 2:**
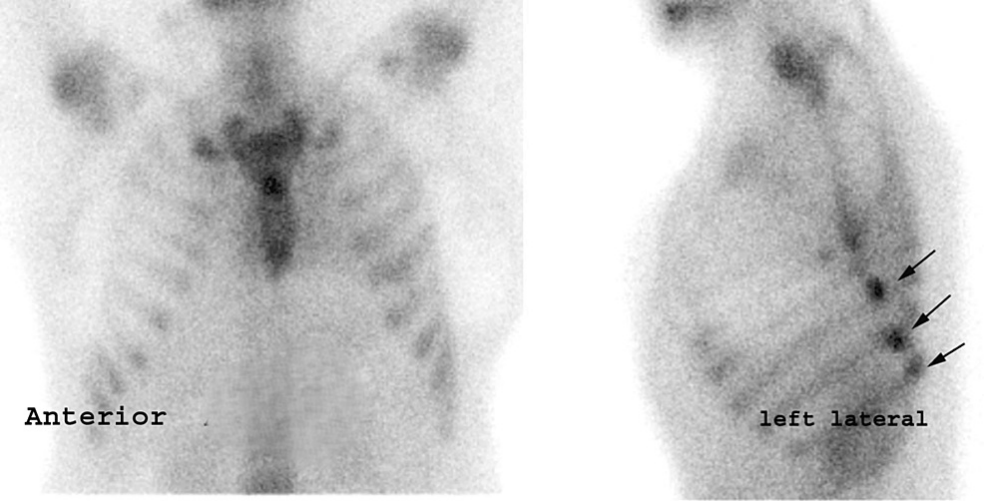
Anterior and left lateral planar Tc-99m PYP scintigraphy Anterior and left lateral planar Tc-99m PYP scintigraphy showing multiple focal rib lesions (arrows), overlapping vertical linear uptake in the left humerus noted superior to the fracture sites. Tc-99m PYP: technetium-99m pyrophosphate

Rib abnormalities on bone scans are common, with a high false positive rate for metastases. Multiple rib lesions are common in patients with multiple bone metastasis, but the rib is an uncommon site for solitary metastasis. Asymmetrically increased uptake in the left or right rib is also uncommon. Solitary rib lesions seen in bone scans are often secondary to trauma or benign pathology, even in patients with underlying malignancy, In such cases, SPECT/CT may provide accurate localization and characterization of equivocal lesions. A solitary rib lesion with mild uptake without bony expansion or cortical or periosteal reaction on CT is suggestive of a benign rib lesion. Multiple rib lesions can be seen in the case of rib fractures or metastasis. The probability that rib lesions detected on bone scans are fractures, rather than metastasis, increases if they are focal, as opposed to linear, and if they decrease in intensity within three to six months or are aligned to involve two or more ribs in the same location (Figure 2) [17]. Pathological rib fracture occurs in the context of pre-existing bone lesions, such as osseous metastatic lesions, multiple myeloma, or lymphoma [18].

There is a high prevalence of degenerative spine disease in patients with suspected cardiac amyloidosis, particularly in elderly patients. The low specificity of bone scintigraphy results in difficult and uncertain interpretations of abnormal uptake in the thoracic spine. Benign processes, such as degenerative diseases, osteoporotic fractures, and primary or metastatic bone diseases, have similar appearances (i.e., increased radiotracer uptake) [19]. A single hot spot in the bone scan is often difficult to categorize in patients with known primary tumors; in a planar image, vertebral lesions are often difficult to localize and characterize [20]. SPECT images are useful in delineating the vertebral body, the pedicles, and the spinous process; for example, a lesion that extends from the vertebral body to the posterior element or that involves the pedicle is more likely to represent metastasis compared with a lesion confined to the facet joints [21]. However, multiple linear abnormalities of varying intensity favor a benign etiology, such as osteoporotic fractures occurring at different time intervals [22].

Soft Tissue Uptake

**Figure 3:**
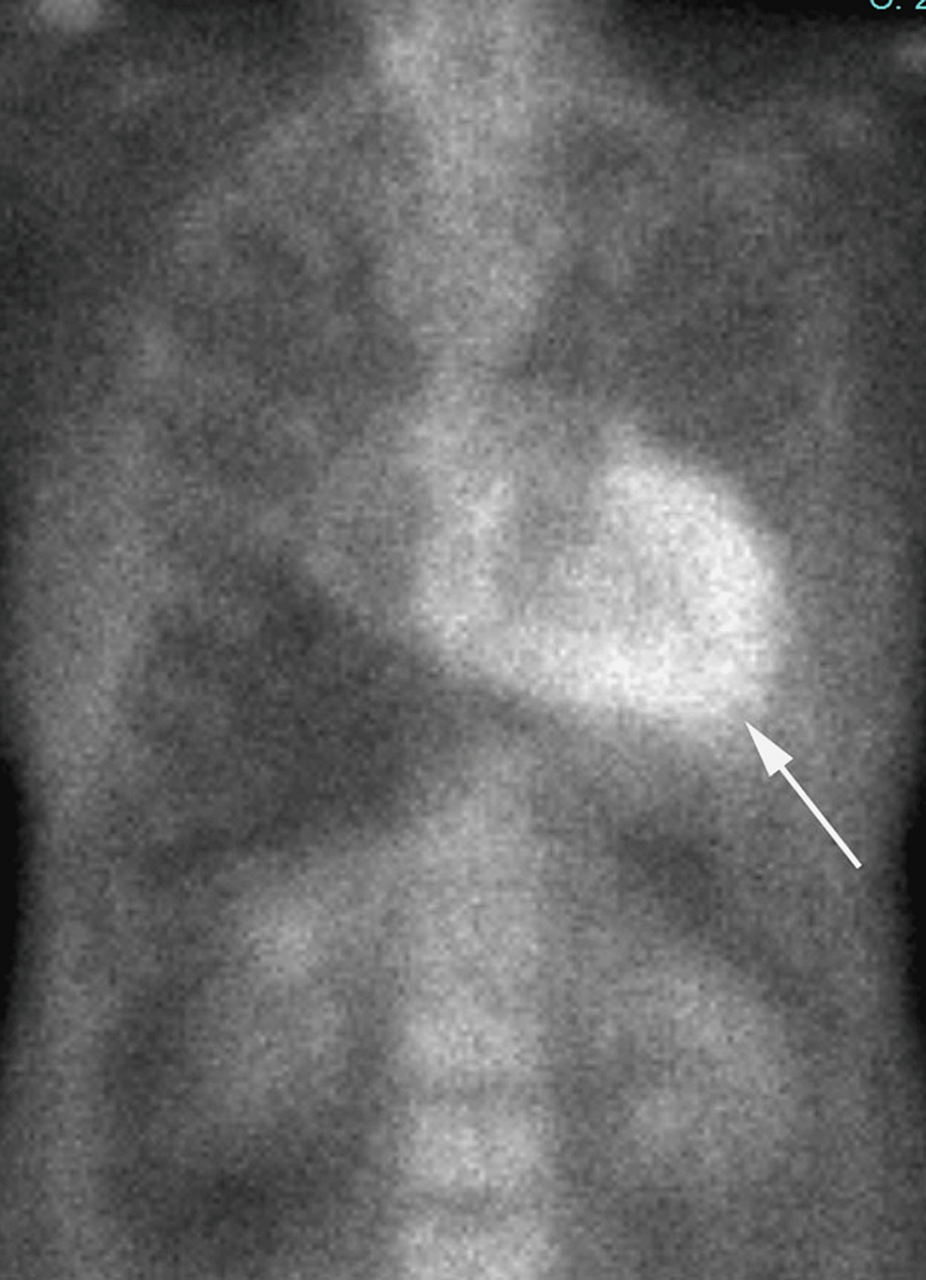
Anterior planar images of Tc-99m PYP scintigraphy Anterior planar images of Tc-99m PYP scintigraphy with intense myocardial uptake of PYP (visual score of 3) and high background activity in a patient with known chronic kidney disease. Tc-99m PYP: technetium-99m pyrophosphate

Tracer uptake in soft tissue may be of diagnostic value in some cases. In general, the mechanism of TC-99m PYP uptake in the soft tissue appears to be similar to that in the bones and involves adsorption into tissue calcium, hyperemia, local tissue necrosis or damage, presence of iron deposits, and altered capillary permeability [23,24]. Generalized increases in soft tissue uptake compared with a normal bone scan can be due to renal failure, dehydration, or a shortened interval between the radiotracer injection and imaging; delayed imaging for three hours is mandatory to obtain better image interpretation and for accurate localization of the tracer (Figure 3).

**Figure 4:**
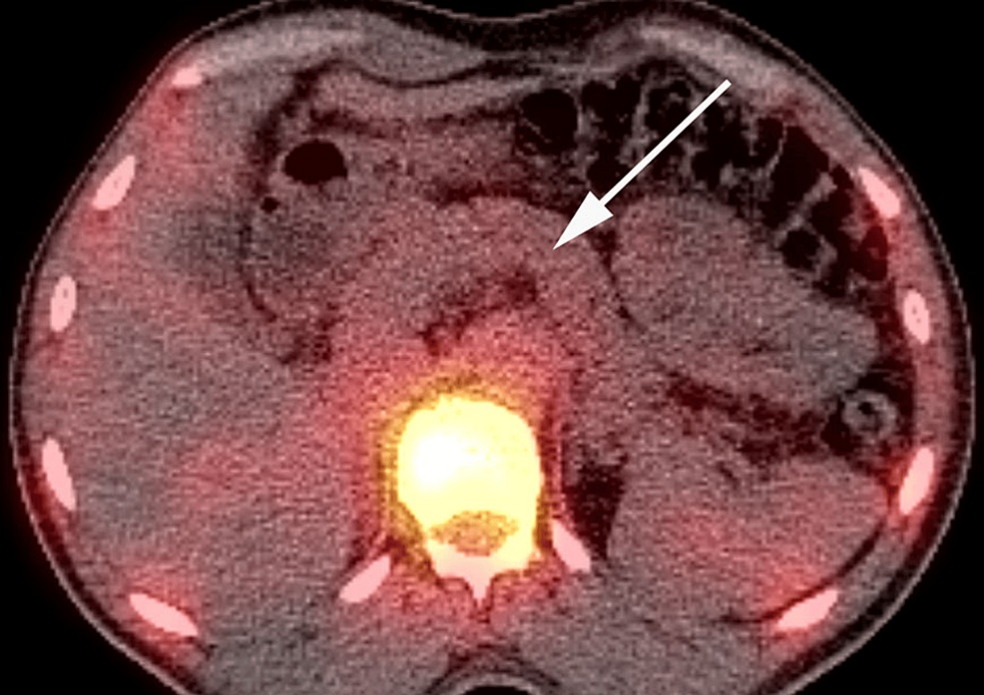
Colored fused SPECT/CT images Anterior planar images of Tc-99m PYP scintigraphy showing absent myocardial uptake (visual score of 0) and an abnormal location of the kidneys, images not shown, Colored fused SPECT/CT images showing a horseshoe kidney. Tc-99m PYP: technetium-99m pyrophosphate; SPECT: single-photon emission computed tomography

**Figure 5:**
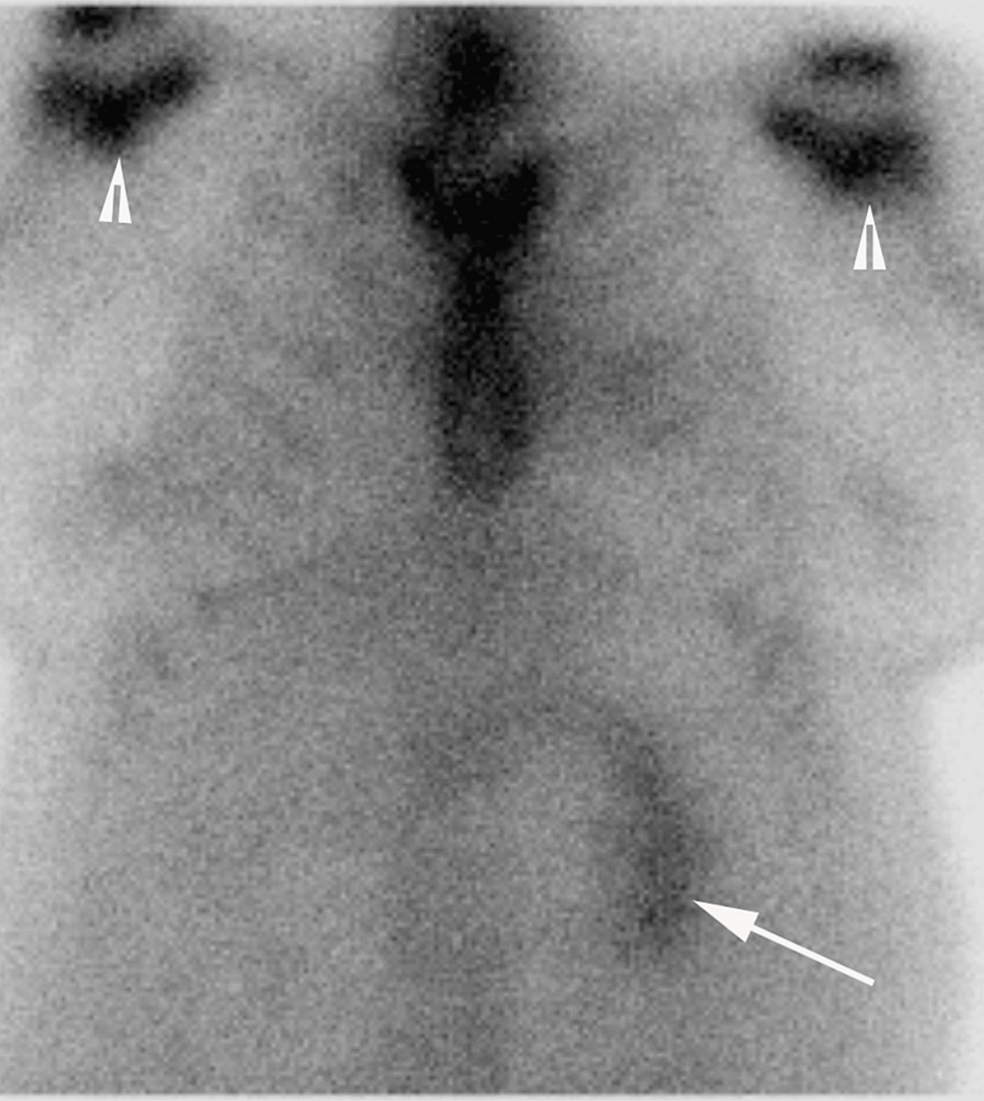
Anterior planar images of Tc-99m PYP scintigraphy Anterior planar images of Tc-99m PYP scintigraphy showing mild bilateral shoulder uptake consistent with degenerative changes (arrowheads), poor visualization of the right kidney, and normal uptake in the left kidney (arrow). Tc-99m PYP: technetium-99m pyrophosphate

In contrast, a generalized decrease in soft tissue uptake compared with a normal bone scan can be due to a prolonged interval between the tracer injection and imaging. Normal structures, such as the kidney and bladder, should be noted; tracer uptake in the kidney can be focal or diffuse. Asymmetrical kidney uptake or absent uptake in one kidney may raise the possibility of abnormal kidney function, and additional evaluation with ultrasound imaging may be warranted; however, these structures are not typically included in the field of view (Figures 4, 5).

**Figure 6:**
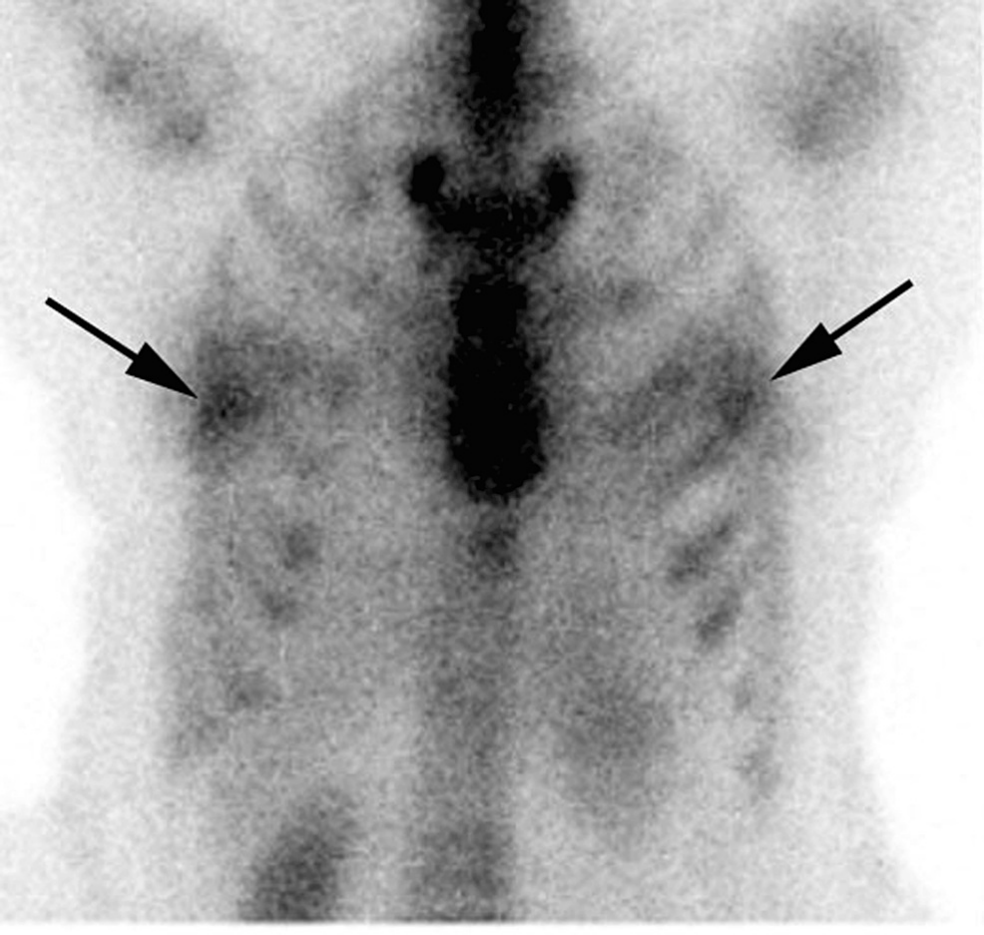
Anterior planar images of Tc-99m PYP scintigraphy Anterior planar images of Tc-99m PYP scintigraphy showing bilateral breast uptake. Tc-99m PYP: technetium-99m pyrophosphate

Pulmonary uptake has been reported in radiation pneumonitis, after radiotherapy, in bronchogenic carcinoma, and in sarcoidosis. An increased PYP in the pleural effusion may indicate a malignancy and pulmonology referral for evaluation is recommended [25,26]. Mild bilateral breast uptake is occasionally seen in a PYP scan as a normal variant. Other causes of diffuse breast uptake of a bone-seeking tracer are gynecomastia and breast cancer in both men and women; focal radiotracer uptake in the breast has also been reported in men and women with breast cancer (Figure 6). Other breast findings that may interfere with the interpretation of planar images include large breast shadows and breast implants; SPECT/CT images are very helpful in such circumstances.

Findings on attenuation-correction CT images

CTAC is frequently used to correct SPECT images and to better localize the tracer uptake activity (e.g., uptake in the myocardium vs. blood pool activity) during TC-99m PYP scanning. Typically, a low-dose CT acquisition is performed through the chest to match the range of the SPECT scan. This low-dose chest CT scan offers detection of several noncardiac findings, both benign and malignant, that may previously be known or unknown. A previous SPECT/CT myocardial perfusion study showed that 70% of myocardial SPECT/CT studies have both minor and major findings on CT images [27]. Most of the extracardiac incidental findings, up to 52.6%, were previously unknown. A similar study reported that 497 (27%) of 1,819 patients enrolled had positive findings on SPECT/CT; of these, 423 (23%) were new, and 51 patients (2.8%) had findings that were considered clinically significant [28]. Although the clinical relevance of extracardiac findings on the TC-99m PYP scan remains under debate, proper recognition of these findings is critical to the comprehensive care of patients.

Musculoskeletal Findings

The prevalence of bone abnormalities on CTAC used for myocardial perfusion imaging (MPI) with SPECT/CT is reported to be 1.9% [29]; these include rib fracture, bone metastasis, vertebral hemangioma, osteophyte formation, degenerative changes, and previous interventions (e.g., vertebroplasty). In general, SPECT/CT has improved the diagnostic accuracy of the bone scan in trauma assessments and explored other musculoskeletal diseases, such as occult fracture, inflammatory arthritis, and spondyloarthropathies [30]. Most of the bone abnormalities detected on CTAC demonstrate TC-99m PYP uptake.

Cardiovascular Findings

**Figure 7:**
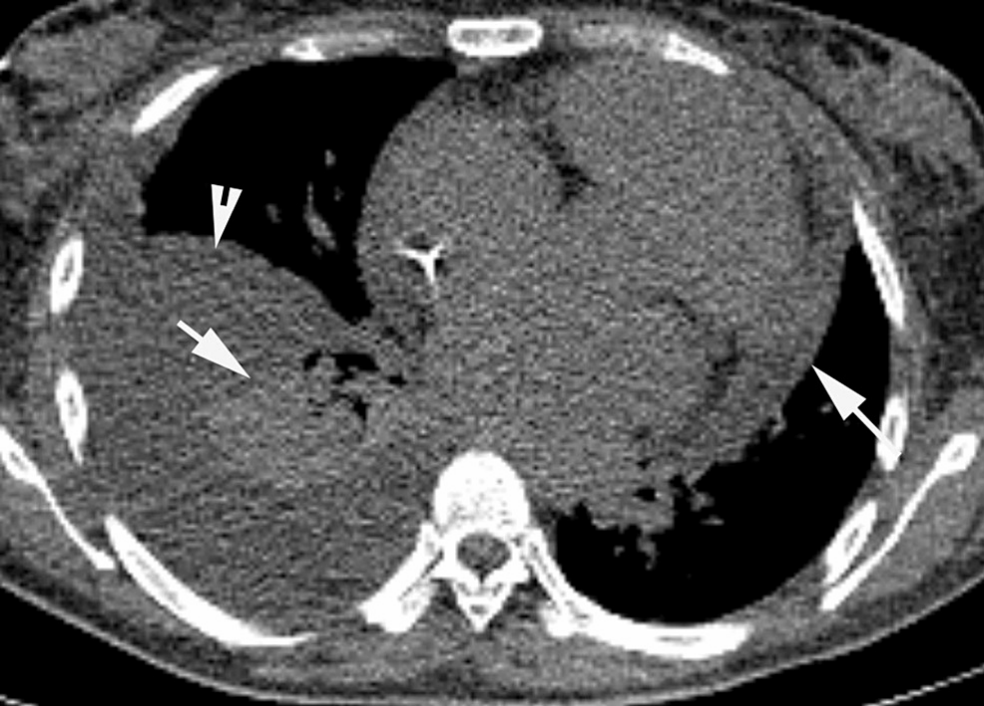
Non-contrast enhanced chest CT Incidental findings on CT images obtained for attenuation correction: pericardial effusion (long arrow), right lung atelectasis (short arrow), and right side pleural effusion (arrowhead)

CTAC images may reveal several cardiovascular abnormalities; one example is coronary artery calcification (CAC), which is relatively common in patients referred to TC-99m PYP scan, as most of these patients are old and have multiple risk factors for coronary artery disease (CAD). Visual scoring of CAC has been reported previously; a patient with severe diffuse CAC on visual exam may have coronary atherosclerosis, so CAD risk stratification is highly recommended. Additional work-up, such as MPI, is recommended if there is high clinical suspicion of CAD. Aortic valve and mitral annular calcifications are common and easily seen on CTAC images. Previous valve interventions, such as transcatheter aortic valve implantation, can be seen on CTAC images (Figure 7).

Other potential findings on CTAC are dilated ascending aorta, pericardial effusion, and cardiomegaly. Several vascular abnormalities can be observed incidentally on CTAC. One example is ascending aortic calcification and dilatation; the prevalence of ascending aortic dilatation (4 cm to 5 cm) is approximately 2.7%. Aortic aneurysm is defined as nonreversible dilation of the ascending aorta to more than 5 cm; these dilations are often atherosclerotic and are commonly seen in elderly patients [31]. Another example is pulmonary artery dilation/dilatation; a normal pulmonary artery size is less than 29 mm in men and less than 27 mm in women. Pulmonary artery dilatation has many causes, but the most common cause is pulmonary hypertension [31].

Thorax

Pulmonary nodules are probably the most common noncardiac CTAC finding that may require additional work-up, such as a diagnostic chest CT. The prevalence of pulmonary nodules in CTAC images on a PYP scan is unknown; however, the prevalence of pulmonary nodules on a CTAC image of an MPI was reported to be 20.6% [32]. The description of the nodule should include an assessment of the size, density, location, and border. Most primary lung cancers have an irregular or spiculated border, whereas pulmonary metastases are usually smooth and multiple and, rarely, can be miliary. Other benign findings included calcified granuloma and hamartoma. Hamartomas present as a coin-shaped solitary lesion with well-defined edges, typically less than 4 cm in diameter with calcification in 25% to 30% of cases. The Popcorn or comma-shaped appearance of calcification is pathognomic for hamartomas. Pulmonary granulomas present with diverse and nonspecific findings on chest CT, with significant overlapping imaging findings among various forms of pulmonary granulomas. Diffuse parenchymal disease, such as emphysema, is commonly observed in patients undergoing cardiac CT. Other diffuse parenchymal disease includes multifocal pneumonia, pulmonary edema, and pulmonary hemorrhage. Bronchiectasis resulting from chronic infection causes localized or diffuse bronchial dilation. Other common benign parenchymal lung abnormalities include fissure opacity or atelectasis, previous lung surgery (e.g., previous lobectomy), or pectus deformity.

Pleural effusion is a common finding detected on CTCA; transudative effusions are commonly caused by left heart failure, but exudative effusions are commonly parapneumonic or related to malignancy. The breasts are typically included in the PYP scan, and a breast lesion found on CTCA must be reported for additional evaluation to rule out cancer. Common mediastinal findings on CTCA include benign and malignant enlargement of the mediastinal nodes; Benign mediastinal node enlargement is typically small with maintained fatty hilum, but malignant nodes have a low-density center, indicating central necrosis, although central necrosis may be seen in other disease such as tuberculosis. Common causes of malignant mediastinal node enlargement are lymphoma and metastases.

Upper Abdomen

**Figure 8:**
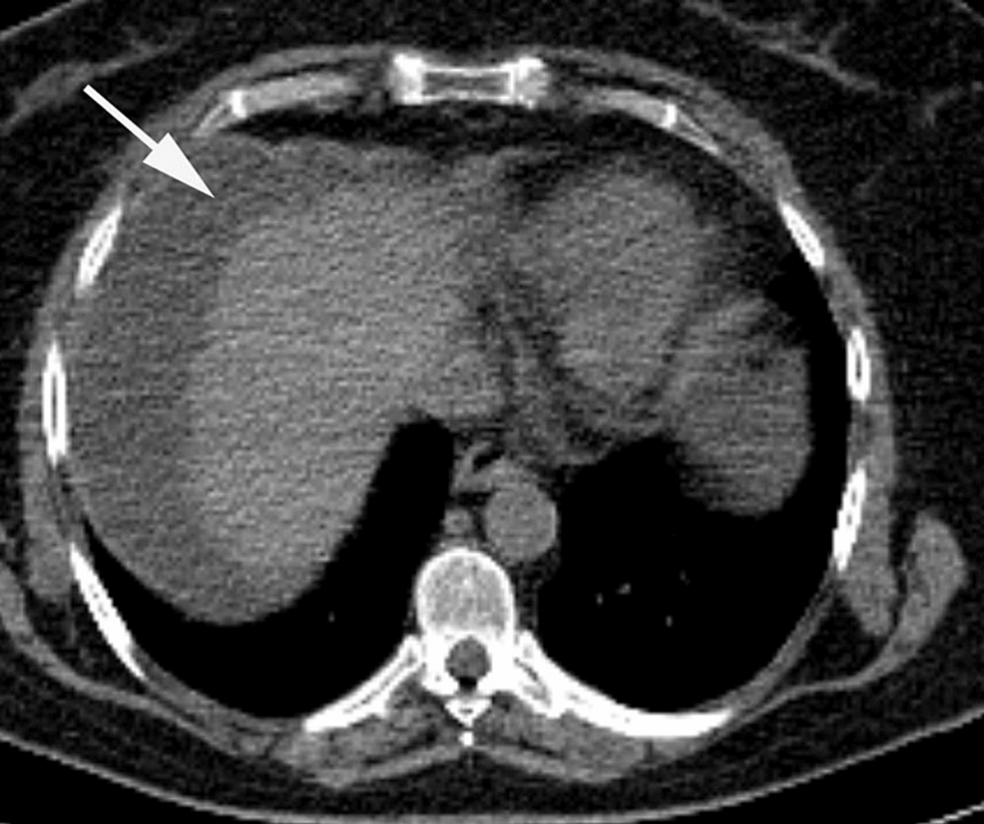
Non-contrast enhanced CT of the upper abdomen Upper abdominal incidental findings on computed tomography CT images, ascites (arrow)

One of the more common and well-known incidental noncardiac findings on CTAC is hiatal hernia; these are present in approximately 10% of the population, and the incidence increases with age [33]. There is no specific follow-up for incidentally detected hiatal hernias. An abdominal aortic aneurysm is defined as the local dilation of the aorta with a maximum diameter of more than 30 mm or more than 1.5 times the diameter of the proximal aorta. Aneurysms smaller than 5.5 cm should be observed with serial abdominal ultrasounds, but aneurysms greater than 5.5 cm are usually considered for surgical or endovascular repair [34]. Several types of liver abnormality can be incidentally detected on CTAC, including nonalcoholic fatty liver disease, liver cirrhosis, and liver tumors. Hepatocellular carcinoma diffluent may be detected with intravenous contrast and other abnormalities that can be seen on CTAC include liver cysts, liver hemangiomas, and gallstones. HCC can have a variety of appearances on CT including focal, multifocal, or diffuse appearance that may be difficult to distinguish from associated cirrhosis. On CT, a hepatic cyst is usually well circumscribed and demonstrates homogenous hypoattenuation around 0-10 Hounsfield unit (HU). Most hepatic hemangiomas are relatively well defined with homogenous hypoattenuation less than 20 HU relative to liver parenchyma. Ascites are a common complication of liver cirrhosis and can be seen on the upper abdomen. The literature has described renal abnormalities, such as renal cysts, renal stones, renal tumors, and hydronephrosis, as well as several splenic lesions, such as splenic cysts, calcifications, and splenomegaly (Figure 8) [29,35-37].

## Conclusions

TC-99m PYP cardiac scan with planar, SPECT, or SPECT/CT is a well-established tool for evaluating patients with suspected cardiac amyloidosis and it has high diagnostic accuracy. Noncardiac findings are commonly encountered in TC-99m PYP studies, some of which can be clinically relevant. Regardless of whether the findings are obvious or subtle, or major or minor, they can impact patient care and follow-up. Detecting and reporting such abnormalities requires careful evaluation of all images, including planar images, rotating maximum intensity projection images, and CT images obtained for attenuation correction. Therefore, interpretation of TC-99m PYP scan images should include both cardiac and noncardiac findings.
